# Taxonomy and SSU rRNA gene-based phylogeny of two new *Euplotes* species from China: *E. chongmingensis* n. sp. and *E. paramieti* n. sp. (Protista, Ciliophora)

**DOI:** 10.1186/s12866-022-02543-9

**Published:** 2022-05-16

**Authors:** Kun Han, Hongbo Pan, Jiamei Jiang

**Affiliations:** 1 Shanghai Universities Key Laboratory of Marine Animal Taxonomy and Evolution, Shanghai Ocean University, Shanghai, 201306 China; 2 Key Laboratory of Exploration and Utilization of Aquatic Genetic Resources, Ministry of Education, Shanghai Ocean University, Shanghai, 201306 China

**Keywords:** Ciliates, Diversity, Estuary, Euplotida, Molecular systematics

## Abstract

**Background:**

The genus *Euplotes* Ehrenberg, 1830, one of the most complicated and confused taxa, contains about 160 nominal species. It was once proposed to be divided into four genera, two of which were proved to be non-monophyletic. At least 19 new species have been discovered in the past decade, implying that there is a large undiscovered diversity of this genus.

**Results:**

The morphology of two new freshwater euplotid ciliates, *Euplotes chongmingensis* n. sp. and *E. paramieti* n. sp., isolated from Shanghai, China, were investigated using live observations, protargol staining, and Chatton-Lwoff silver staining method. *Euplotes chongmingensis* is characterized by its small size (40–50 × 25–35 μm), about 24 adoral membranelles, 10 frontoventral cirri, two marginal and two caudal cirri, eight dorsolateral kineties with 11–16 dikinetids in the mid-dorsolateral kinety and a double type of silverline system. *Euplotes paramieti* n. sp. is 180–220 × 110–155 μm in vivo and strongly resembles *E. amieti* but having a difference of 57 bp in their SSU rRNA gene sequences. Phylogenetic analyses based on SSU rRNA gene sequence data were used to determine the systematic positions of these new taxa.

**Conclusions:**

The description of two new freshwater taxa and their SSU rRNA gene sequences improve knowledge of biodiversity and enrich the database of euplotids. Furthermore, it offers a reliable reference for environmental monitoring and resource investigations.

## Background

Ciliates are a diverse group of unicellular eukaryotic organisms that are widely studied in ecological, environmental, evolutionary, and basic biological research [1–4]. The genus *Euplotes* Ehrenberg, 1830, is widely distributed in marine, freshwater, and terrestrial habitats and contains about 160 nominal species [5–8]. *Euplotes* species circumscription and identification are mainly based on the habitat, the body shape and size, the shape of the adoral zone of membranelles, the ventral ciliary pattern, the number of dorsolateral kineties, the nuclear apparatus and the type of silverline system [7, 9–11]. However, some species are highly similar or overlap in morphological features. Therefore, molecular information, mainly the small subunit ribosomal RNA (SSU rRNA) gene sequence, has been widely used to help identify or distinguish cryptic species [12–14]. In addition, genomes from at least four *Euplotes* species have been sequenced, which may be useful for the identification of new *Euplotes* species[15–18].

Borror and Hill [19] split *Euplotes* into four genera: *Euplotes*, *Euplotopsis*, *Euplotoides*, and *Moneuplotes*, based on characteristics of cortical structure, endosymbionts, morphometric data, morphogenetic patterns, and ecology. This classification has repeatedly been disclaimed by genetic analyses which show that neither *Euplotes* nor *Euplotopsis* is monophyletic [20, 21]. Nevertheless, reliable subdivisions have yet to be discovered, therefore most taxonomic studies still recognize *Euplotes* sensu Ehrenberg, 1830, as we do in the present study.

There is increasing evidence that the biogeographical distribution of ciliates follows the moderate endemicity model [22, 23], which indicates there are unknown species in underexplored habitats. This assertion is supported by the discovery in China in the past decade of eight new *Euplotes* species isolated from previously unsampled habitat or areas [24–28]. Yangtze estuary is an example of a location that has been rarely sampled for its ciliate biodiversity. In the present study, two novel euplotid ciliates were isolated from this estuary. Both were found to be new species of *Euplotes*. Each is described based on its morphology and SSU rRNA gene sequence following the recommendations in Warren et al. [29]. Their molecular phylogeny was analyzed to determine their evolutionary relationships.

## Results

**Spirotrichea Biitschli, 1889**

**Euplotida Small & Lynn, 1985**

***Euplotes*** ***Ehrenberg, 1830***

### *Euplotes chongmingensis* n. sp. (Figs. 1 and 2, Table 1)

**Fig. 1:**
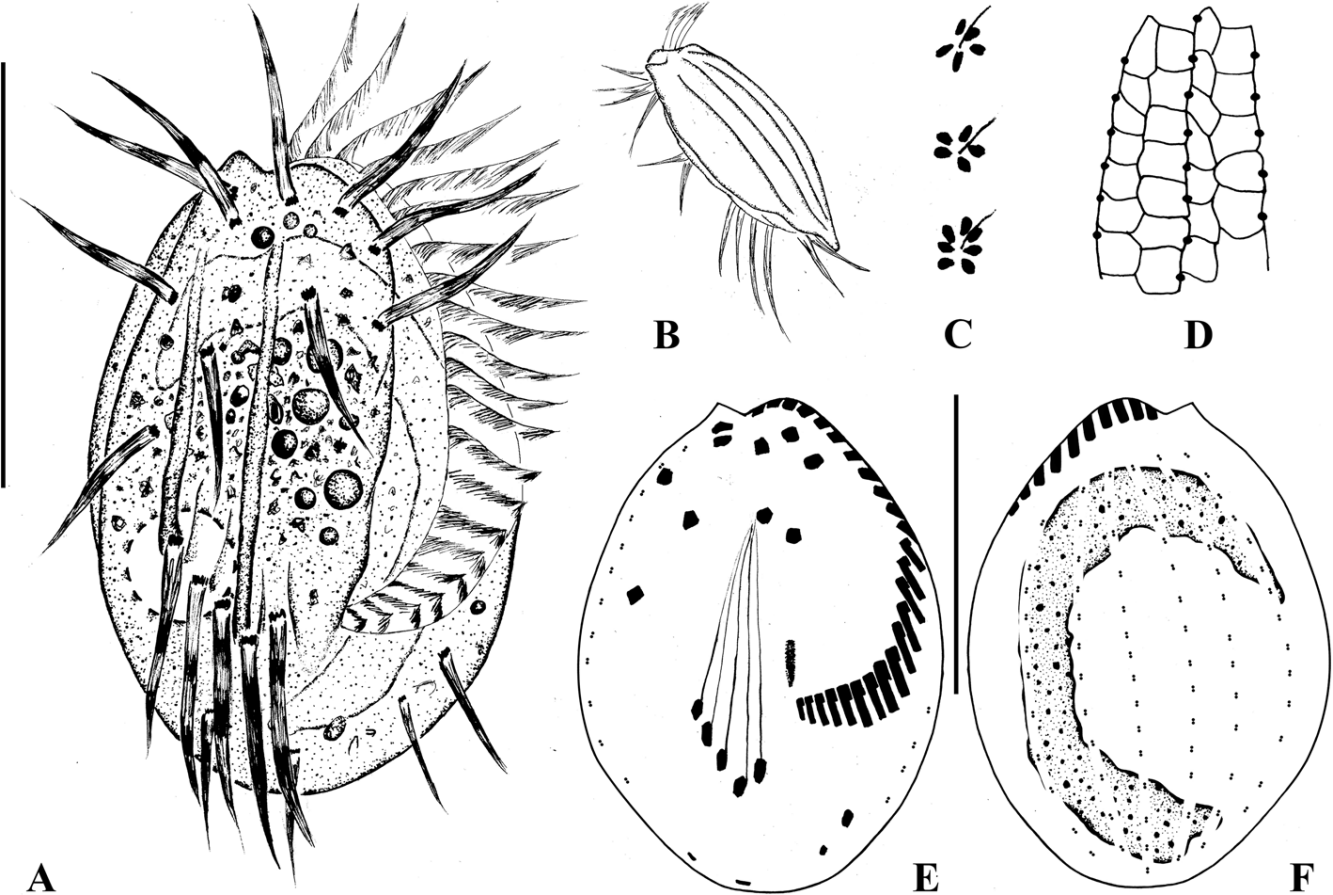
*Euplotes chongmingensis* n. sp. in vivo (**A**–**C**), after silver nitrate staining (**D**) and after protargol staining (**E**, **F**). **A **Ventral view to show a representative individual. **B** Lateral view. **C** Detail of dorsal side showing cortical ampules surrounding the cilia. (**D**) Detail of dorsal silverline system. **E**, **F** Ventral (**E**) and dorsal (**F**) views of the holotype specimen showing the ciliary pattern, the fibrils, and the macronucleus after protargol staining. Scale bars = 30 μm

**Fig. 2:**
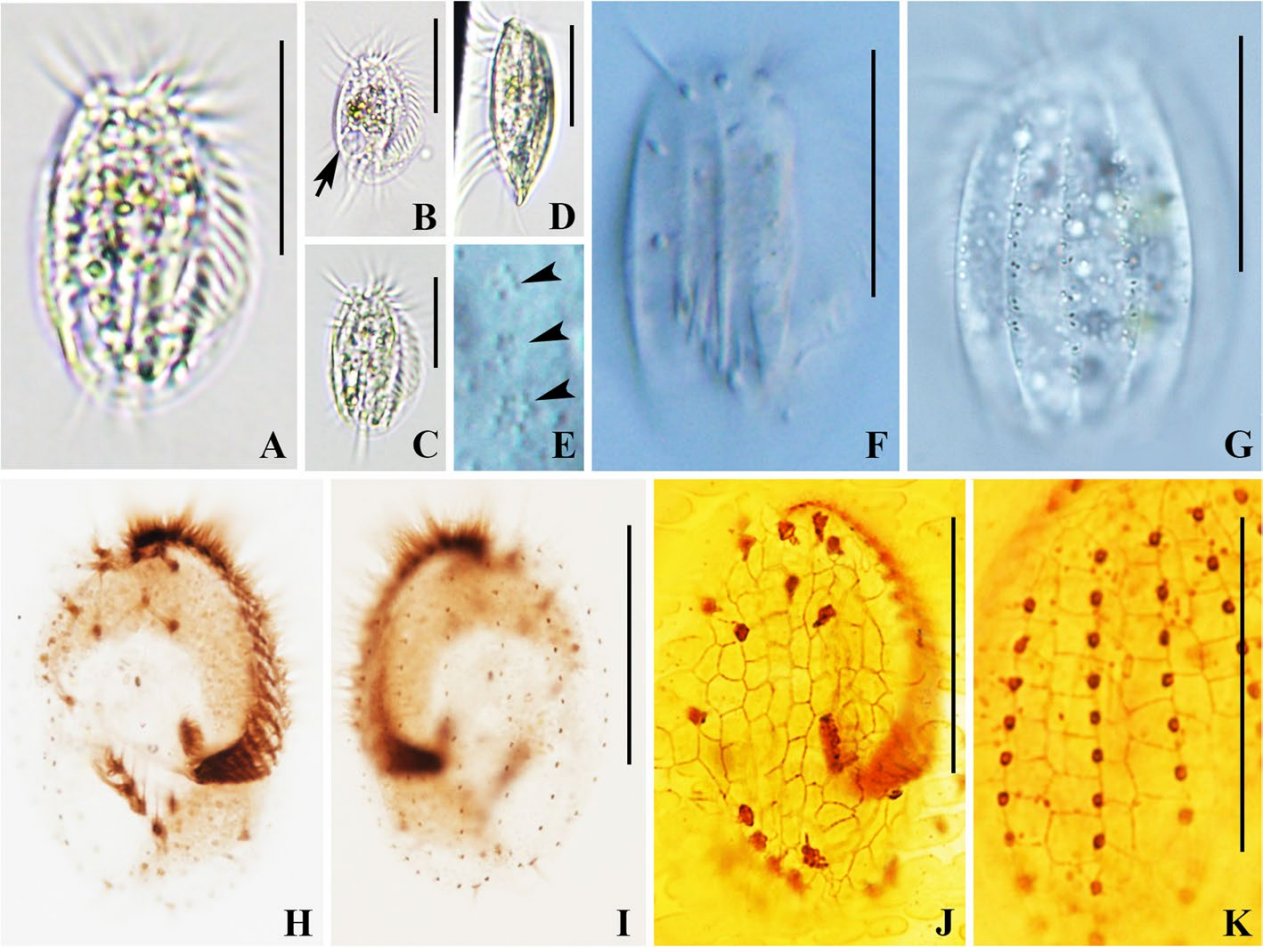
*Euplotes chongmingensis* n. sp. in vivo (**A**–**G**), after protargol staining (**H**, **I**) and after silver nitrate staining (**J**, **K**). A Ventral view to show representative individual in vivo. **B**, **C** Ventral views, to show different body shapes, arrow points to the contractile vacuole. **D** Lateral view. **E** Detail of dorsal side showing the cortical ampules surrounding the cilia. **F** Ventral view showing the longitudinal ridges. **G** Dorsal view showing the dorsal ridges. **H**, **I** Ventral (**H**) and dorsal (**I**) views of the holotype specimen showing the ciliary pattern and the macronucleus. **J**, **K** Ventral (**J**) and dorsal (**K**) silverline system. Scale bars = 30 μm

**Table 1:** Morphometric data of *E. chongmingensis* n. sp. based on protargol-stained specimens

Character	Min	Max	Mean	Median	SD	SE	CV	*n*
Body length (μm)	40	54	44.5	46.0	10.1	2.3	22.8	20
Body width (μm)	21	41	31.9	32.0	4.6	1.0	14.4	20
Adoral membranelles, number	22	26	23.9	24.0	1.2	0.3	5.1	20
Length of adoral zone (μm)	26	34	30.0	30.0	2.3	0.5	7.6	20
Frontoventral cirri, number	10	10	10.0	10.0	0	0	0	20
Transverse cirri, number	5	5	5.0	5.0	0	0	0	20
Marginal cirri, number	2	2	2.0	2.0	0	0	0	20
Caudal cirri, number	2	2	2.0	2.0	0	0	0	20
Dorsolateral kineties, number	8	8	8.0	8.0	0	0	0	20
Dikinetids in mid-dorsolateral kinety, number	11	16	12.4	12.0	1.2	0.3	9.9	20
Dikinetids in leftmost kinety, number	3	5	3.7	4.0	0.7	0.1	17.8	20

*CV* coefficient of variation in %, *Max* maximum, *Mean* arithmetic mean, *Min* minimum, *n* number of cells measured, *SD* standard deviation, *SE* Standard error

#### Diagnosis

Freshwater *Euplotes*, about 40–50 × 25–35 μm in vivo; buccal field about 75% of cell length with about 24 membranelles; five conspicuous dorsal ridges; cirrotype-10 pattern; five transverse cirri; two marginal cirri, two caudal cirri; six dorsal kineties and two other kineties that run laterally along sides of body, with 11–16 dikinetids in mid-dorsolateral kinety; macronucleus C-shaped; dorsal silverline system double type.

#### Type specimens

The protargol slide with the holotype specimen (No. HK2020100901-1) and a Chatton-Lwoff silver nitrate slide with paratype specimens (No. HK2020100901-2) are deposited in the Laboratory of Protozoology, Shanghai Ocean University.

#### Type locality

A river on Chongming Island, Shanghai, China (31°50′32.6"N, 121°16′02.5"E), where the temperature was 17.3 °C and salinity was 0‰.

#### Etymology

The species-group name *chongmingensis* refers to the area (Chongming Island) where the sample was collected.

ZooBank registered details of *Euplotes chongmingensis* n. sp.: urn:lsid:zoobank.org:act:A7ACE2C2-46E8-4CD6-92E7-4D74D443C493

#### Morphological description

Cell size usually 40–50 × 25–35 μm in vivo, and 40–54 × 21–41 μm (on average 46.4 × 31.9 μm) after protargol staining, length to width ratio about 1.8:1 in live cells, about 1.5:1 in protargol-stained specimens. Dorsoventrally flattened about 1:3 (Figs. 1B, 2D). Cell shape generally oval in outline, left and right margins convex, anterior end narrowly rounded with a distinct projection on right side (Figs. 1A, 2A, B and C). Five ventral ridges between transverse cirri, three short, two long and conspicuous (Figs. 1A, 2F). Five longitudinal ridges on dorsal side (Fig. 2G). Five to seven cortical ampules (about 1.0 × 0.7 μm) surrounding each dorsal bristle-like cilium (Figs. 1C, 2E). Cytoplasm colorless, with opaque endoplasmic particles in mid-body region (Figs. 1A, 2A, B and C). Contractile vacuole 7 μm across, located at right posterior (Fig. 2B), pulsating at intervals of about 35 s. Macronucleus typically C-shaped; micronucleus not observed (Figs. 1F, 2I). Locomotion usually by fast crawling and jerking movements.

**Fig. 3:**
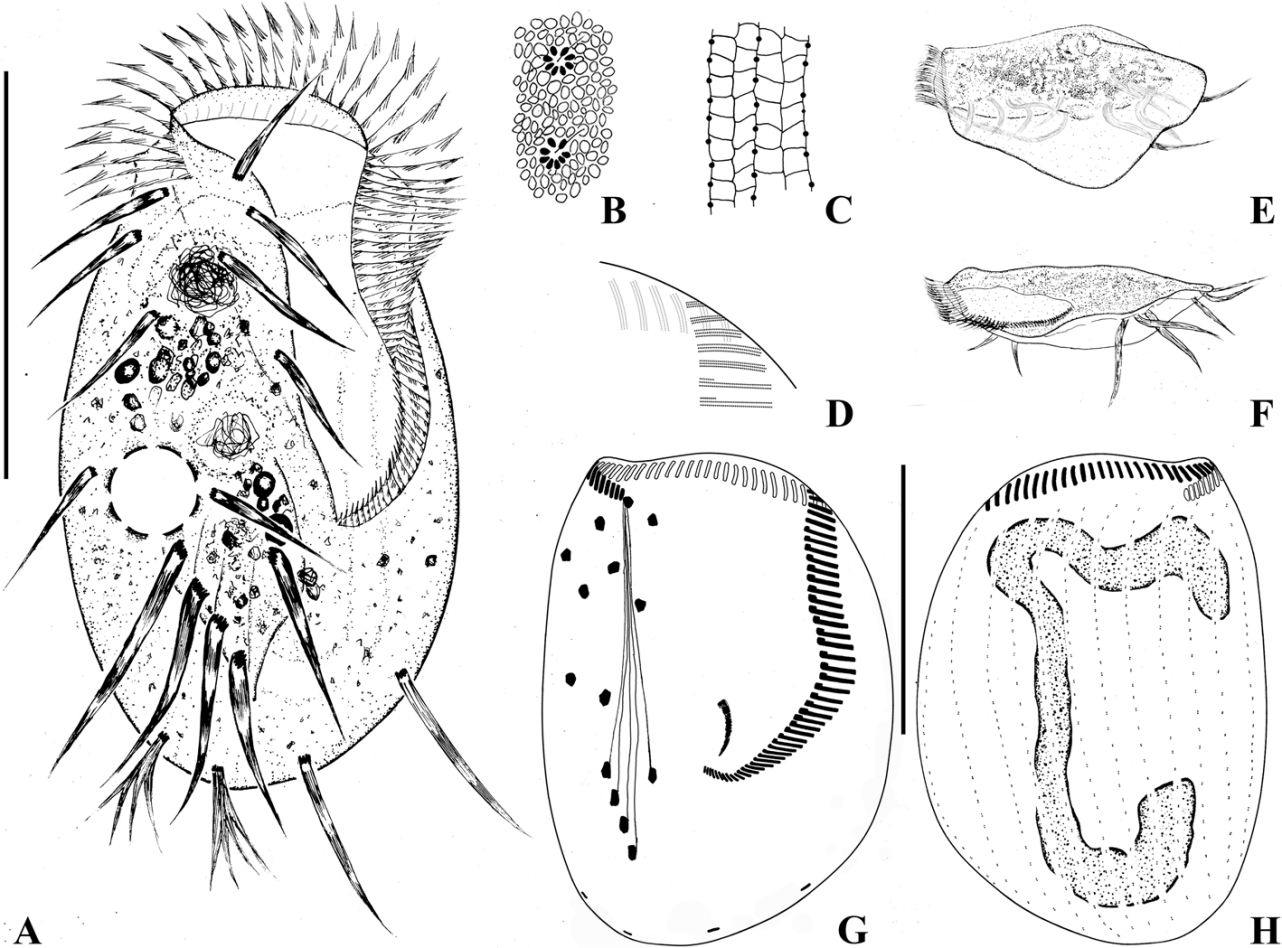
*Euplotes paramieti* n. sp. in vivo (**A**, **B**, **E**, **F**), after protargol (**C**, **D**, **G**, **H**) and silver nitrate (**C**) staining. A Ventral view to show a representative individual. **B** Detail of dorsal side showing the cortical ampules (shaded black) surrounding the cilia and numerous irregular ellipsoidal to ovoidal granules extremely densely packed beneath dorsal pellicle (**C**) Detail of dorsal silverline system. **D** Structure of anterior left membranelles. E, F Left lateral views of a ‘winged’ (**E**) and a normal (**F**) individual. **G**, **H** Ventral (**G**) and dorsal (**H**) views of the holotype specimen showing the ciliary pattern, fibrils, and macronucleus. Scale bars = 90 μm

**Fig. 4:**
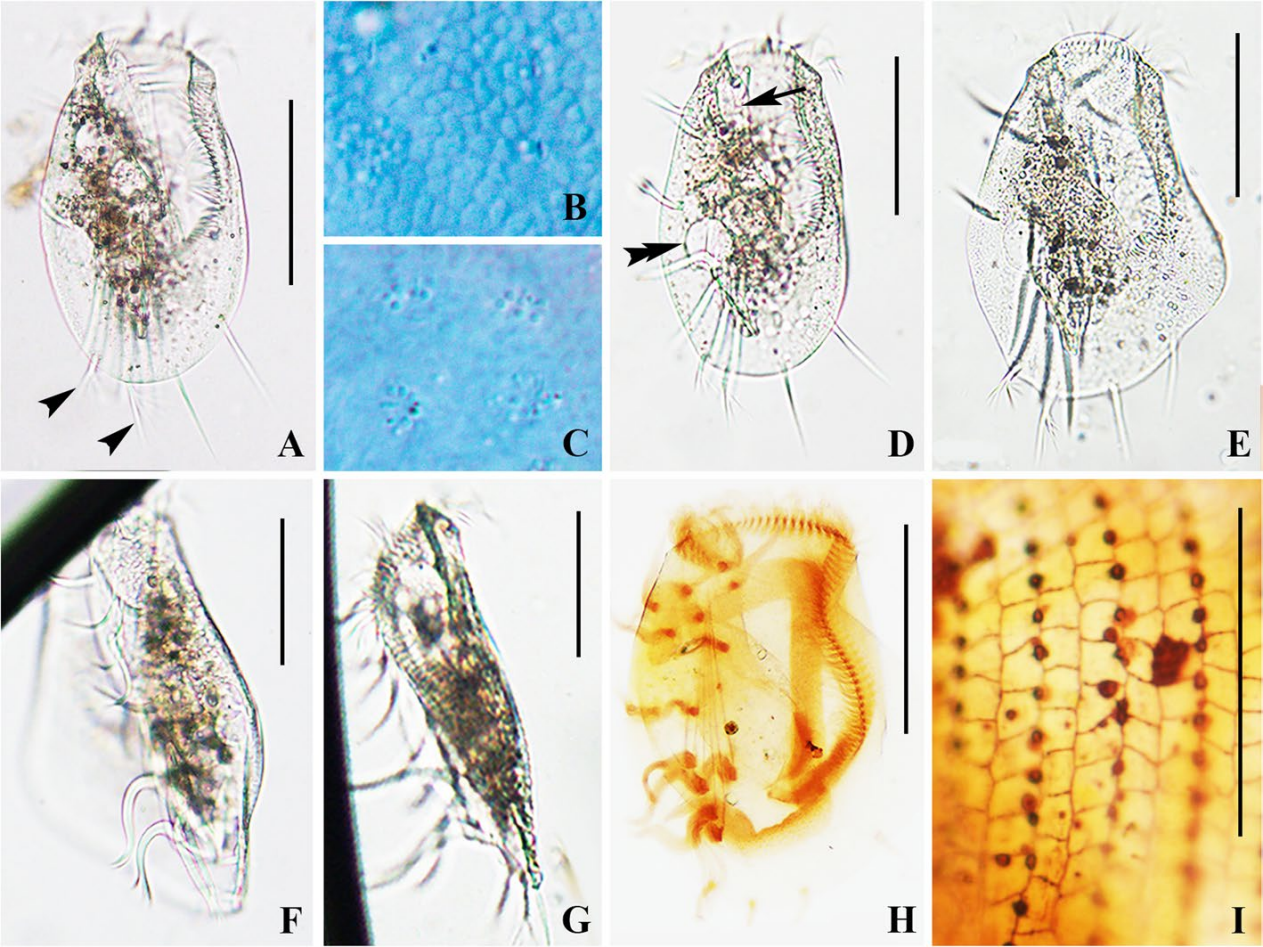
*Euplotes paramieti* n. sp. in vivo (**A**–**G**), after protargol (**H**) and silver nitrate (**I**) staining. **A**, **D**, **E** Ventral views, to show different body shapes. Arrowheads show distal end of each caudal cirrus is forked, arrow shows the pouch along right side of peristome, double arrowhead points to the contractile vacuole. **B** Numerous irregular ellipsoidal to ovoidal granules extremely densely packed beneath dorsal pellicle. **C** Detail of dorsal side showing the cortical ampules surrounding the cilia. **F**, **G** Left lateral views of ‘winged’ (**F**) and normal (**G**) individual. **H** Ventral view showing the ciliary pattern and the macronucleus. **I** Detail of dorsal silverline system. Scale bars = 90 μm

**Fig. 5:**
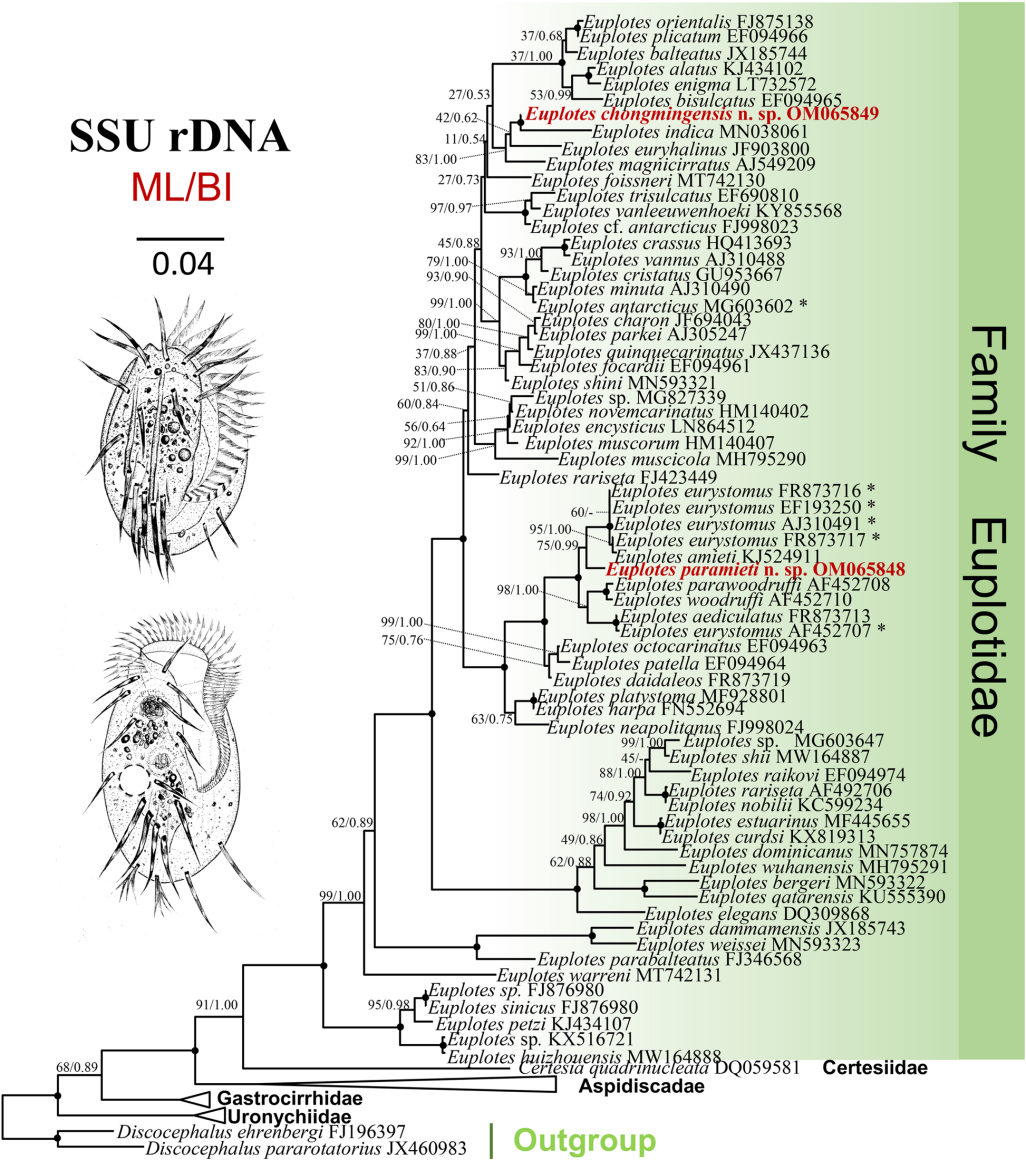
The maximum likelihood (ML) tree inferred from SSU rDNA sequences, showing the phylogenetic positions of *Euplotes chongmingensis* n. sp. and *E. paramieti* n. sp. (bold, red font). Numbers at nodes represent the bootstrap values of the ML analysis and the posterior probability of the Bayesian inference method. ‘-’ indicates difference in topology between the ML and BI phylogenies, ‘*’ indicates doubtful species identity. Fully supported (100ML/1.00 BI) branches are marked with solid circles. The scale bar corresponds to four substitutions per 100 nucleotide positions. All branches are drawn to scale

Adoral zone prominent, extending to 75% of body length, evenly curved, composed of 22–26 membranelles. Paroral membrane short, about 6 × 1 μm, composed of many irregularly arranged kinetosomes, lying to right of posterior portion of adoral zone (Fig. 1E). Ten frontoventral cirri, about 16 μm long in vivo; five transverse cirri, about 18 μm long, arranged V-shaped pattern; two marginal cirri, about 13 μm long, lower one much smaller than upper one; two caudal cirri, about 13 μm long (Table 1 and Figs. 1A, E, 2A, H). Eight dorsolateral kineties with 11–16 dikinetids in mid-dorsolateral kinety (Figs. 1F, 2I), 3–5 dikinetids in leftmost kinety on ventral side (Figs. 1C, 2H). In three of 11 specimens observed, very few polygons between adjacent dorsal kineties are of nearly equal width, but we can still clearly classify them as double type based on structure of most polygons (Figs. 1D, 2K).

### *Euplotes paramieti* n. sp. (Figs. 3 and 4, Table 2)

**Table 2:** Morphometric data of *E. paramieti* n. sp. based on protargol-stained specimens

Character	Min	Max	Mean	Median	SD	SE	CV	*n*
Body length (μm)	157	201	182.9	184.0	14.8	4.4	8.1	20
Body width (μm)	123	158	141.5	143.0	13.7	4.1	9.7	20
Adoral membranelles, number	63	93	77.4	80.0	9.9	3.0	12.8	20
Length of adoral zone (μm)	118	135	126.3	128.0	6.4	1.9	5.1	20
Frontoventral cirri, number	9	9	9.0	9.0	0	0	0	20
Transverse cirri, number	5	5	5.0	5.0	0	0	0	20
Marginal cirri, number	2	2	2.0	2.0	0	0	0	20
Caudal cirri, number	2	2	2.0	2.0	0	0	0	20
Dorsolateral kineties, number	12	13	12.2	12.0	0.2	0	1.3	20
Dikinetids in mid-dorsolateral kinety, number	24	37	29.5	29.0	17.5	1.3	59.3	20
Dikinetids in leftmost kinety, number	14	23	18.3	17.0	7.0	0.8	38.4	20

*CV* coefficient of variation in %, *Max* maximum, *Mean* arithmetic mean, *Min* minimum, *n* number of cells measured, *SD* standard deviation, *SE* Standard error

#### Diagnosis

Freshwater *Euplotes*, about 180–220 × 110–155 μm in vivo; Buccal field broad and prominent, with 63–93 membranelles, ventral part sigmoidal; no conspicuous ridges; cirricirrotype-9 pattern I, five transverse cirri, leftmost one nearly at same level as rightmost one; two marginal cirri; two caudal cirri; usually 12 dorsolateral kineties with 24–37 dikinetids in mid-dorsolateral kinety; macronucleus 3-shaped; dorsal silverline system double type.

#### Type specimens

The protargol slide with the holotype specimen (No. HK2020102802-1) and a Chatton-Lwoff silver nitrate slide with paratype specimens (No. HK2020102802-2) are deposited in the Laboratory of Protozoology, Shanghai Ocean University.

#### Type locality

Intertidal zone from Hengsha Island, Shanghai, China (31°18′17.8''N, 121°49′49.7''E), where the temperature was 18.8 °C and salinity was 0.25‰.

#### Etymology

The species-group name *paramieti* is a composite of the prefix *para-* (Greek preposition, beside, like) and the species-group name *amieti*, referring to the similarity of this species to *Euplotes amieti*.

ZooBank registered details of *Euplotes paramieti *n. sp.: urn:lsid:zoobank.org:act:0FB8427F-718F-41E3-ABDC-DA44BD71B9AB

#### Morphological description

Cells in vivo about 180–220 × 110–155 μm. Body usually oval in outline, right margin more convex than left, posterior end generally broadly rounded (Figs. 3A, 4A, D). About half of cells observed presented a conspicuous lateral “wing” on left margin (Figs. 3E, 4E, F). Body dorsoventrally flattened about 3.5: 1 (Figs. 3F, 4G). Buccal field dominant, length about 60% of cell length. Conspicuous collar positioned at anterior end (Fig. 3A, D, E). Deep pouch along right side of peristome (Figs. 3A, 4A, D, E). No obvious ridges on ventral or dorsal side. Eight or nine cortical ampules (2 × 1 μm) surround each dorsal cilium (Figs. 3B, 4C). Numerous ellipsoidal granules densely arranged beneath dorsal pellicle (Figs. 3B, 4C). Cytoplasm colorless, with opaque food vacuoles and numerous densely packed particles in central part making this region dark grey whereas other regions are highly transparent (Figs. 3A, 4A, D and F). Contractile vacuole about 25 μm across, located 60% down length of body near right margin (Figs. 3A, 4D). Locomotion typically by moderately fast crawling or slight jerking or swimming in the water while rotating about the longitudinal axis of body. Resting cysts not observed.

Adoral zone prominent, composed of 63–93 membranelles. Distal portion on ventral side curved, followed by 20 membranelles on dorsal side, each comprising three rows of equal length (Figs. 3A, G, H, 4D, E). Proximal ventral membranelles arranged in sigmoidal shape, majority comprising one short and two longer rows (Figs. 3A, G, H, 4H). Paroral membrane about 20 × 4 μm, composed of many irregularly arranged kinetosomes, positioned below buccal lip, extending to proximal end of adoral zone (Figs. 3G, 4H).

Nine frontoventral cirri, about 35 μm long in vivo, cirrus VI/3 lacking; five strong transverse cirri, about 50 μm long, four cirri on right arranged in oblique line, clearly separated from leftmost cirrus; rightmost and leftmost cirri nearly at same level (Figs. 3A, G, 4H); two marginal cirri located near left margin of body, about 40 μm in length. Two caudal cirri, each about 25 μm long and with a forked distal end. Twelve or 13 (usually 12) dorsolateral kineties extending almost entire length of body, 24–37 dikinetids in the mid-dorsolateral kinety, 14–23 dikinetids in leftmost kinety on ventral side. Macronucleus 3-shaped (Figs. 3H, 4H). Dorsal silverline system double type (Figs. 3C, 4I).

### Systematic positions of two new species based on SSU rRNA gene sequences

The length, GC content and GenBank accession number of the SSU rRNA gene of *E. chongmingensis* and *E. paramieti* are 1,869 bp, 44.35%, OM065849, and 1,878 bp, 44.46%, OM065848, respectively.

Phylogenetic trees inferred by ML and BI analyses had similar topologies, therefore only the ML is shown with nodal support from both algorithms (Fig. 5). Euplotida and each of its five families, namely Aspidiscidae, Certesiidae, Euplotidae, Gastrocirrhidae and Uronychiidae, is monophyletic. *Euplotes chongmingensis* clusters with its morphologically similar species *E. indica* with maximal support (100% ML, 1.00 BI), and then clusters successively with *E. euryhalinus* (JF903800) and *E. magnicirratus* (AJ549209). The sequence similarities of *E. chongmingensis* with each of these three species are 96.83%, 97.12% and 97.16%, respectively.

*Euplotes paramieti* clusters with *E. eurystomus* (FR873716, EF193250, AJ310491, FR873717) and *E. amieti* (KJ524911) forming a clade that is sister to the *E. woodruffi* (AF452710) + *E. parawoodruffi* (AF452708) + *E. aediculatus* (FR873713) + *E. eurystomus* (AF452707) clade with maximal support.

## Discussion

### *Euplotes chongmingensis* n. sp.

Five freshwater species should be compared with *E. chongmingensis* n. sp. as each has a similar-size body, 10 frontoventral cirri, two marginal cirri, and a double silverline system. These are: *E. indica* Abraham et al., 2021, *E. vanleeuwenhoeki* Serra et al., 2020, *E. palustris* Hagen, 1980*, E. wuhanensis* Lian et al., 2019*,* and *E. crenosus* Tuffrau, 1960 (Table 3).

**Table 3:** Comparison of *E. chongmingensis* n. sp. with its related congeners with cirrotype-10, two marginal cirri and a double type silverline system

Characteristics	*E. chongmingensis*	*E. palustris*	*E. wuhanensis*	*E. crenosus*	*E. vanleeuwenhoeki*	*E. indica*
Cell size in vivo (μm)	40–50 × 25–35	45–55 × 35–45	40–50 × 25–30	50–70	49.1 ± 4.7 × 32.7 ± 3.8	49–52 × 40–46
Habitat	freshwater	freshwater	soil	freshwater	freshwater	freshwater
No. of AM	22–26	25–35	18–24	25–30	22–29	20–25
No. of DK	8	8	7	8	7–8	7
No. of DK_mid	11–16	11–14	7–12	ca. 23^b^	13–14	11–16
Macronucleus	C-shaped	C-shaped^b^	C-shaped	C-shaped	C-shaped or 3-shaped	C-shaped
Special features	lower marginal cirrus conspicuously small	cirri IV/2 and V/2 small; transverse cirri in two groups	cirri V/2 and VI/2 highly reduced; indistinct gap separating transverse cirri into two groups	^a^	Three dorsal longitudinal furrows	^a^
Data source	Present paper	[32]	[24]	[33]	[31]	[30]

*AM* adoral membranelles, *MC* marginal cirri, *CC* caudal cirri, *DK* dorsolateral kineties, *DK*_*mid* dikinetids in mid-dorsal kinety

^a^No special features

^b^Counted based on the illustration

Abraham et al. [30] discovered *E. indica* from Raj Ghat pond in India. It is very similar with *E. chongmingensis* n. sp. in terms of its cell length in vivo (49–52 μm vs. 40–50 μm), the C-shaped adoral zone of membranelles and the number of adoral membranelles (20–25 vs. 22–26). However, it differs from *E. chongmingensis* n. sp. in having a wider body in vivo (40–46 μm vs. 25–35 μm), fewer dorsal ridges (5 vs. 6), and fewer dorsolateral kineties (7 vs. 8) [30]. The SSU rRNA gene sequence of the type population of *E. indica* (MN038061) has a 96.83% similarity with *E. chongmingensis*, and thus is the adelphotaxon of the latter.

*Euplotes vanleeuwenhoeki* was found in Kolleru Lake, India by Serra et al. [31]. It resembles *E. chongmingensis* in cell size and the pattern of both the ventral and dorsal infraciliature. However, *E*. *vanleeuwenhoeki* has three longitudinal furrows on the dorsal surface that reach the posterior region of the cell [31]*.* Furthermore, these two species are placed in different clades in the SSU rRNA gene tree and they have a sequence dissimilarity of 3.99%.

*Euplotes palustris* was reported by Hagen [32] from marshy areas in Westfalen, Germany. It resembles *E. chongmingensis* n. sp. in cell size (45–55 × 35–45 μm in vivo) and dorsal ciliature. However, it can be separated from the new species by having two distinctly weaker frontoventral cirri IV/2 and V/2 (vs. 10 normal-sized frontoventral cirri in *E. chongmingensis* n. sp.) and its transverse cirri are arranged in two groups (vs. in one group) [32]. Molecular information for *E palustris* has not been reported.

*Euplotes wuhanensis* was isolated from Luojia Hill, Wuhan, China by Lian et al. [24]. It resembles *E. chongmingensis* n. sp. in cell size (40–50 × 25–30 μm vs. 40–50 × 25–35 μm in vivo) and the number of adoral membranelles (18–24 vs. 22–26 in *E. chongmingensis* n. sp.). However, it differs from *E. chongmingensis* n. sp. in having two conspicuously smaller frontoventral cirri V/2 and VI/2, fewer dorsolateral kineties (7 vs. 8), and fewer dikinetids in the mid-dorsal kinety (7–12 vs. 11–16). In addition, it has five transverse cirri arranged sparsely, with an indistinct gap between the left two and the three right cirri (vs. cirri arranged in a tick-shaped row) [24]. Furthermore, *E. wuhanensis* (MH7095291) is genetically distinct from *E. chongmingensis*, with an SSU rRNA gene sequence similarity of 85.41%.

*Euplotes crenosus* was first isolated by Tuffrau [33] in France, and has never been redescribed. It resembles *E. chongmingensis* n. sp. in the ventral infraciliature and nuclear apparatus. However, it can be separated from *E. chongmingensis* n. sp. by having a longer body (50–70 μm vs. 40–50 μm in vivo), an almost straight (vs. sharply curved) adoral zone of membranelles, and more dikinetids in the mid-dorsal kinety (ca. 23 vs. 11–16) [33]. No molecular information is available for *E. crenosus*.

### *Euplotes paramieti* n. sp.

#### Wing structure

Wings have been reported in populations of *Euplotes muscicola* Kahl, 1932, *E. lynni* Abraham et al. 2021, *E. aediculatus* Pierson, 1943*, E. octocarinatus* Carter, 1972, *E. patella* Ehrenberg, 1838, and *E. novemcarinata* Wang, 1930 [11, 30, 49–52]. It has been shown that some freshwater *Euplotes* can change their morphology, for example by developing wings or a dorsal keel, to defend themselves against the risk of predation [49]. In our non-clonal cultures of *E. paramieti* n. sp., *Daphnia* sp., which is considered a potential predator of ciliates [53], was present. The wing in *E. paramieti* n. sp. may therefore be a predator-induced defense response caused by the presence of *Daphnia* rather than being a diagnostic character for species circumscription and identification.

#### Comparison of *Euplotes paramieti* n. sp. with other species

In terms of its freshwater habitat, large body size, two marginal cirri, and cirrotype-9 pattern I, there are five species that should be compared with *E. paramieti*: *E. amieti* Dragesco, 1970, *E. aediculatus* Pierson, 1943, *E. eurystomus* Wrześniowski, 1870, *E. woodruffi* Gaw, 1939 and *E. octocarinatus* Cater 1972 (Table 4).

**Table 4:** Comparison of *E. paramieti* n. sp. with freshwater congeners that share a large-sized body, cirrotype-9 pattern I, two marginal cirri and a double silverline system

Characteristics	*E. paramieti*	*E. amieti*	*E. eurystomus*	*E. aediculatus*	*E. woodruffi*	*E. octocarinatus*
Cell size in vivo (μm)	180–220 × 110–155	130–240 × 70–160	88–180 × 40–135	105–170 × 60–120	83–200 × 58–130	60–126 × 31‒76
No. of AM	63–93	52–70	44–65	40–60	51–85	30–42
No. of DK	12–13 (12)	12–15	8–12	8–9	9–11	8
No. of DK_mid	24–37	18–32	15–25	18–26	17–35	12–19
Macronucleus	3-shaped	3-shaped	3-shaped	C- to 3-shaped	T- or Y-shaped	C-shaped
Data source	Present paper	[5, 34–37]	[6, 38–41]	[7, 30, 33, 42–44]	[5, 12, 30, 38, 42, 45–47]	[7, 48]

*AM* adoral membranelles, *DK* dorsolateral kineties, *DK*_*mid* dikinetids in mid-dorsal kinety

*Euplotes amieti* has been reported several times, i.e., from Cameroon, Rwanda, Canada and Shanghai, China [8, 40–43]. It strongly resembles *E. paramieti* in living morphological characteristics and ciliary pattern, however it can be separated from the latter by having fewer adoral membranelles (52–70 vs. 63–93) and in the arrangement of the transverse cirri (in a tick-shaped row vs. the leftmost cirrus nearly at the same level as the rightmost cirrus) [8, 40–43]. The SSU rRNA gene sequence of *E. amieti* Shanghai population (KJ524911) differs from that of *E. paramieti* by 57 bp with a dissimilarity of 2.63%.

*Euplotes eurystomus* was originally reported from Poland by Wrześniowski [41] and has since been discovered in India, America, Italy, Japan, and China [30, 39, 40, 54–56]. The five SSU rRNA gene sequences of *E. eurystomus* (AF452707, AJ310491, EF193250, FR873716, FR873717) in GenBank are not identical and none of them is associated with reliable morphological data implying that misidentifications may have occurred, so this species needs to be reinvestigated. Nevertheless, the *E. eurystomus-*complex can be separated from *E. paramieti* n. sp. by its smaller body size in vivo (88–180 × 40–135 μm vs. 180–220 × 110–155 μm), and in having fewer adoral membranelles (44–65 vs. 63–93), dorsolateral kineties (8–12 vs. 12–13) and dikinetids in the mid-dorsal kinety (15–25 vs. 24–37) [6, 38–41].

*Euplotes aediculatus* was first discovered by Pierson [42] in USA and has since been reported from Europe, North America, Africa, New Zealand, Antarctica, India, and China [36, 43, 57, 58]*.* It differs from *E. paramieti* n. sp. in having a smaller size in vivo (105–170 × 60–120 μm vs. 180–220 × 110–155 μm), the presence (vs. absence) of dorsal ridges, the appearance of the adoral zone of membranelles (curved in a C-shape vs. somewhat 3-shaped), the number of adoral membranelles (40–60 vs. 63–93), the arrangement of the transverse cirri (in a tick-shaped row vs. leftmost cirrus at almost the same level as the rightmost cirrus), and in having fewer dorsolateral kineties (8–9 vs. 12–13) and fewer dikinetids in the mid-dorsal kinety (18–26 vs. 24–37) [36, 43, 57, 58]. *Euplotes aediculatus* (FR873713) is genetically distinct from *E. paramieti* n. sp. with an SSU rRNA gene sequence dissimilarity of 2.93%.

*Euplotes woodruffi* was originally isolated by Gaw [59] from a freshwater pond in Wuhan, China, and has since been found in both freshwater and brackish water habitats [5, 12, 30, 36, 38, 42, 45–47, 56, 60]. All these populations were reported to be *E. woodruffi*, except the two descriptions reported by Song and Bradbury [47] and Shen et al. [60], in which *E. woodruffi* was renamed *E. parawoodruffi* which was later confirmed to be a junior synonym of *E. woodruffi* [12]. *Euplotes woodruffi* differs from *E. paramieti* n. sp. by its T- or Y-shaped (vs. 3-shaped) macronucleus, the presence (vs. absence) of conspicuous dorsal ridges, smaller body size in vivo (83–200 × 58–130 μm vs. 180–220 × 110–155 μm), and in having fewer adoral membranelles (51–85 vs. 63–93), dorsolateral kineties (9–11 vs. 12–13) and dikinetids in the mid-dorsal kinety (17–35 vs. 24–37) [5, 12, 30, 38, 42, 45–47]. Genetically, *Euplotes woodruffi* (AF452710) and *E. parawoodruffi* (AF452708) are distinct from *E. paramieti*, with an SSU rRNA gene sequence dissimilarities of 2.71% and 2.66%, respectively.

*Euplotes octocarinatus* was first described by Carter [7] from Lake Wingra spillway, Madison, USA. It differs from *E. paramieti* n. sp. by its smaller body size in vivo (60–126 × 31‒76 μm vs. 180–220 × 110–155 μm), the presence (vs. absence) of dorsal ridges, the appearance of the adoral zone of membranelles (evenly curved vs. somewhat 3-shaped), the arrangement of the transverse cirri (in a tick-shaped row vs. the leftmost cirrus nearly at the level of the rightmost cirrus), and in having fewer adoral membranelles (30–42 vs. 63–93), dorsolateral kineties (8 vs. 12–13), and dikinetids in the mid-dorsal kinety (12–19 vs. 24–37) [10]. Furthermore, *E. octocarinatus* (EF094963) is genetically distinct from *E. paramieti*, with an SSU rRNA gene sequence similarity of 95.71%.

#### Phylogenetic analyses

The phylogenetic trees (ML and BI) are consistent with previous phylogenetic analyses, even with the addition of two new taxa [24, 25, 30, 37, 61, 62]. The 10 frontoventral cirri and double dorsal silverline pattern are possibly an ancestral character of euplotids considering the shared traits of the basal clade [30, 63]. Clades within the SSU rRNA gene tree that are supported by a common morphological trait include: one clade comprising species that lack the V/2 cirrus, i.e., species formerly classified as *Euplotides* plus *E. paramieti* n. sp.; and another clade that comprises species with a single dorsal silverline system, i.e., species formerly classified as *Monoeuplotes*, but excluding *E. antarcticus* sensu Liu et al., 2020 (MG603602) which was misidentified [13].

*Euplotes chongmingensis* clusters with *E. indica,* its most similar freshwater species, with full support (100% ML, 1.00 BI), although they have a SSU rRNA gene sequence dissimilarity of 3.17%. The other two most closely related species, *E euryhalinus* and *E. magnicirratus*, are marine, Moreover, their sequences differ from *E. chongmingensis* by 53 bp and 52 bp, respectively. Therefore, validity of *E. chongmingensis* as a separate species is supported.

*Euplotes paramieti* clusters with *E. amieti* (KJ524911) and four populations of *E. eurystomus* (FR873716, EF193250, AJ310491, FR873717). The nucleotide difference between *E. paramieti* and each of these four populations is 41–47 bp. There is another population of *E. eurystomus* (AF452707) in the sister clade. However, the five populations of *E. eurystomus* lack reliable morphological descriptions and/or vouchered specimens, therefore their identity cannot be confirmed.

## Conclusions

We described two novel ciliates from Shanghai, China: *Euplotes chongmingensis* n. sp. and *E. paramieti* n. sp. from River Yangtze estuary, China. The validity of each is supported both by their morphology and their molecular sequences. Some morphological characteristics of the species in the genus *Euplotes* overlap, so using a multidisciplinary approach could reduce the confusion and ambiguity. Some “well-known” species, e.g., *E. eurystomus,* should be reinvestigated considering the questionable sequences in GenBank.

## Methods

### Sample collection and identification

*Euplotes chongmingensis* was collected on 26th Sept. 2019 from a river (31°50′32.6"N, 121°16′02.5"E) on Chongming Island, Shanghai, China, where the water temperature was 17.3 °C and the salinity was 0‰. *Euplotes paramieti* was collected on 28th Oct. 2020 from the intertidal zone of Hengsha Island (31°18′17.8"N, 121°49′49.7"E), at the estuary of the River Yangtze, Shanghai, China, where the water temperature was 18.8 °C and the salinity was 0.25‰. Non-clonal cultures were established and maintained at room temperature (about 20 °C) in Petri dishes containing mineral water with rice grains added to enrich the growth of bacteria as a food source for the ciliates. Ciliate cells were observed in vivo using bright field and Nomarski differential interference contrast microscopy at magnifications between 100 × and 1000 × [64]. The infraciliature and nuclear apparatus were revealed by protargol staining [65] and the silverline systems were revealed by the Chatton–Lwoff silver nitrate staining method [8]. Counts and measurements were performed at a magnification of 1000 × . Drawings of stained specimens were made with the help of a drawing attachment and photomicrographs. Terminology is mainly according to Curds [6], except for the marginal cirri.

### DNA extraction, PCR amplification, and gene sequencing

To avoid contamination, a single cell was picked out using a sterile micropipette and washed five times with distilled water. Genomic DNA was extracted using the DNeasy Blood & Tissue Kit (Qiagen, CA) following the manufacturer’s instructions but modified such that one quarter of the volume of each reagent was used. PCR amplifications of the SSU rRNA gene were performed with the primers 18S-F (5’-AACCTGGTTGATCCTGCCAGT-3’) and 18S-R (5’-TG ATCCTTCTGCAGGTTCACCTAC-3’) [66]. Cycling parameters were as follows: initial denaturation at 98 °C for 30 s, 34 cycles of amplification (98 °C, 10 s; 69 °C, 30 s; 72 °C, 1 min), with a final extension of 72 °C for 5 min. PCR product purification and clone sequencing were performed by Sangon Biotech (Shanghai) company.

#### Phylogenetic analyses

The SSU rRNA sequences of the two new species were aligned with 80 other related ciliate sequences obtained from the National Center for Biotechnology Information (NCBI) database, including all the isolates of *Euplotes eurystomus* (for GenBank accession numbers, see Fig. 5). *Discocephalus ehrenbergi* (JX460983) and *D. pararotatorius* (FJ19639) were chosen as the outgroup taxa. Sequences were aligned using the muscle algorithm [67] in MEGA X [68] with the default parameters. The ends of the resulting alignment were refined by Gblocks (http://www.phylogeny.fr/one_task.cgi?task_type=gblocks), yielding an alignment of 1623 characters.

Maximum likelihood (ML) analysis with 1,000 bootstrap replicates was performed using RAxML-HPC2 on XSEDE 8.2.12 [69] on the CIPRES Science Gateway with the GTRGAMMA model (http://www.phylo.org). Bayesian inference (BI) analysis was applied on the same platform using MrBayes 3.2.7 on XSEDE [70] on the CIPRES Science Gateway with the best fit model GTR + I + G, which was selected by the Akaike Information Criterion (AIC) in MrModeltest 2.2 [71]. Markov chain Monte Carlo (MCMC) simulations were run for 1,000,000 generations with sampling every 100 generations and a burn-in of 1,000 trees. Tree topologies were manually formatted with Figtree 1.4.3 (http://tree.bio.ed.ac.uk/software/Figtree/).

## Data Availability

The datasets used and/or analyzed during the current study are available from the corresponding author on reasonable request.

## Abbreviations

PCR: Polymerase chain reaction
SSU rRNA: Small subunit ribosomal RNA
ML: Maximum likelihood
BI: Bayesian inference
GC: Guanine and cytosine
